# Microfluidic platforms for monitoring cardiomyocyte electromechanical activity

**DOI:** 10.1038/s41378-024-00751-z

**Published:** 2025-01-09

**Authors:** Wei Wang, Weiguang Su, Junlei Han, Wei Song, Xinyu Li, Chonghai Xu, Yu Sun, Li Wang

**Affiliations:** 1 School of Mechanical Engineering, Qilu University of Technology (Shandong Academy of Sciences), 250353 Jinan, China; 2 Shandong Institute of Mechanical Design and Research, 250353 Jinan, China; 3 Department of Minimally Invasive Comprehensive Treatment of Cancer, Shandong Provincial Hospital Affiliated to Shandong First Medical University, 250021 Jinan, China; 4 Department of Mechanical and Industrial Engineering, University of Toronto, Toronto, ON M5S3G8 Canada

**Keywords:** Microfluidics, Biosensors

## Abstract

Cardiovascular diseases account for ~40% of global deaths annually. This situation has revealed the urgent need for the investigation and development of corresponding drugs for pathogenesis due to the complexity of research methods and detection techniques. An in vitro cardiomyocyte model is commonly used for cardiac drug screening and disease modeling since it can respond to microphysiological environmental variations through mechanoelectric feedback. Microfluidic platforms are capable of accurate fluid control and integration with analysis and detection techniques. Therefore, various microfluidic platforms (i.e., heart-on-a-chip) have been applied for the reconstruction of the physiological environment and detection of signals from cardiomyocytes. They have demonstrated advantages in mimicking the cardiovascular structure and function in vitro and in monitoring electromechanical signals. This review presents a summary of the methods and technologies used to monitor the contractility and electrophysiological signals of cardiomyocytes within microfluidic platforms. Then, applications in common cardiac drug screening and cardiovascular disease modeling are presented, followed by design strategies for enhancing physiology studies. Finally, we discuss prospects in the tissue engineering and sensing techniques of microfluidic platforms.

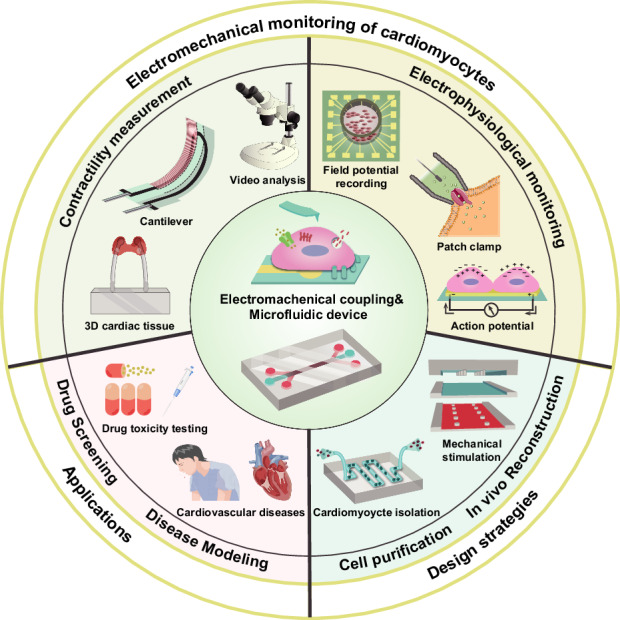

## Introduction

**Fig. 1:**
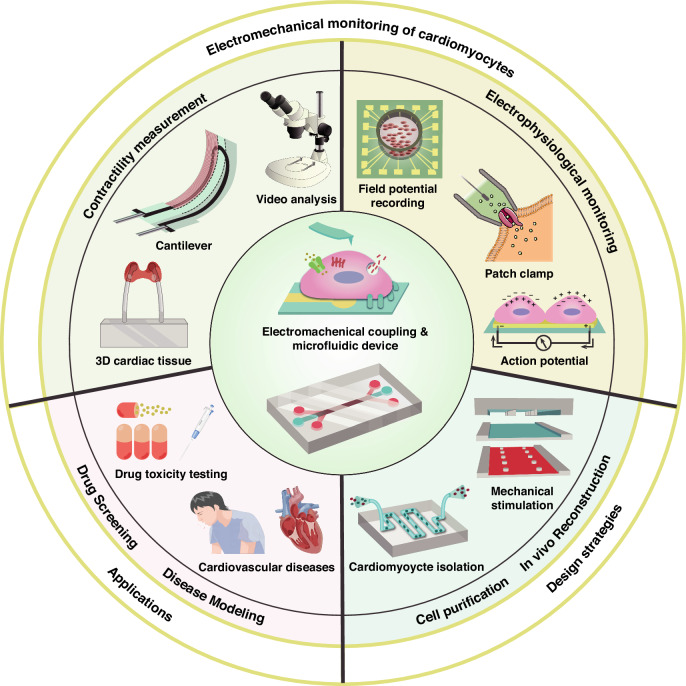
Schematic diagram of the microfluidic platforms used for physiological studies of cardiomyocytes. The main contents comprise contractility and electrophysiological signal detection, its applications, and design strategies

Cardiovascular diseases (CVDs) are the leading cause of death worldwide, accounting for ~40% of all deaths and causing ~17.9 million deaths annually^1,2^. Statistics indicate that the average period to launch a new cardiovascular drug is 10–15 years. Research and development costs range from more than $1 billion to less than $2 billion, and the number is growing at an annual rate of ~8.5%^3,4^. A 90% failure rate in drug development occurs in the clinical trial phase^5^. Animal models are frequently used as subjects for preclinical efficacy assessments in drug development and are capable of providing toxicity and safety screening in the preclinical phase for the clinical prediction of drugs^6^. However, animal models usually struggle to predict clinical efficacy effectively due to differences in pharmacokinetics and underlying molecular mechanisms of the same disease^7^. For this reason, many investigators and pharmaceutical companies have focused on studies with cultured human cells, including primary cells, established cell lines, derivatives of induced pluripotent stem cells (iPSCs), and, more recently, (Fig. 1) human organoids^8^.

Microfluidics can be used to develop and investigate in vitro cell models and manipulate small amounts of fluid at the micron scale. It originates from microanalytical methods such as gas chromatography (GPC) and capillary electrophoresis (CE)^9^. Microfluidic platforms provide diverse cell cultures and biomimetic in vivo environments, which also revolutionize chemical analysis with the advantages of high-throughput and high-sensitivity analysis methods, multiplexing, and easy technical integration for drug screening and disease modeling^10^. Moreover, microfluidic analysis methods can decouple several key physiological features, such as the flow rate, shear stress, and diffusion of liquids^11^. From the perspective of cellular growth and development, the expression levels of cardiac genes under microfluidic conditions surpass those in conventional static two-dimensional culture systems^12^. To this end, microfluidic culture is more compatible with the in vivo environment than conventional culture methods. Moreover, long-term culture and independent monitoring of multiple types of cells, such as cardiomyocytes, liver, intestinal, and cancer cells, are allowed, where heterogeneous responses can be observed^13,14^.

Microfluidics can achieve the stable and controllable high-throughput screening of multiple drugs at multiple concentrations in vitro and can be used to assess the individual or combined effects of two drugs simultaneously^15^. In addition, it can reproduce physiological changes under combined mechanical and chemical stimulus conditions^16^. Moreover, microfluidics is considered a powerful tool for disease modeling and has advantages in its well-defined structure and replication of the extracellular microenvironment^17^. For example, a microfluidic device was used to construct pathological cardiac hypertrophy disease models by simulating mechanical overload through cyclic stretching. This model was used to study disease pathology through the characterization of gene expression, cell morphology, calcium processing, and contractile function^18^.

The electromechanical detection and multiparameter analysis of cardiomyocyte activity are allowed in microfluidics. Specifically, alterations in the contractile strength of cardiomyocytes reflect drug regulation or pathological changes. Electrophysiological signals can detect abnormal heart activity and some diseases that cause arrhythmia, such as atrial fibrillation, stroke, and heart failure^19^. Most electrical, electrophysiological, and optical methods for cell detection can be integrated into microfluidic systems^20,21^. For example, a microfluidic device incorporating an electrode array and lactate microbiosensor could achieve the electrochemical and optical monitoring of metabolites from single cardiomyocytes^22^. The contraction of a single cell analyzed by optical imaging demonstrated the accumulation of byproducts of metabolites. The calcium transients and pH values were recorded independently using fluorescent indicator dyes. Microfluidic devices integrated with patch clamp arrays feature high-density patch sites, side capillaries for capture, and tight electrical seals^23^. Compared with traditional patch clamp technology, the chamber volume was decreased by 10,000 times, which enabled the exposure of attached cells to different reagents with very little reagent consumption and a uniform solution content.

This paper aims to review the methods and technologies applied in microfluidic platforms for monitoring cardiomyocyte electromechanical activity, which is different from previous recent reviews concerning cardiovascular disease research^24^, drug sensing and screening^25^, and fabrication methods and materials of microfluidic devices^26^. In this review, the first chapter introduces both nonmicrofluidic and microfluidic-based platforms for mechanical and electrophysiological signal detection in cardiomyocytes, summarizing existing research findings and classifying them according to technical principles. The second chapter focuses on the applications of microfluidic methods in drug screening, followed by common cardiovascular disease models. Finally, we summarize the design strategies aimed at promoting the study of the physiology of microfluidic platforms. The outcome of this review will contribute to a comprehensive summary of sensing platforms and their applications.

## Contractility measurement technologies

Excitation‒contraction coupling (ECC) is an intracellular signal transduction process mediated by Ca^2+^ that links membrane depolarization and cell contraction^27^. Excitation propagates along the cell membrane in the form of an action potential, triggering membrane depolarization. Then, membrane depolarization causes the opening of L-type calcium channels. When calcium ions enter the cell, they induce the sarcoplasmic reticulum to release more calcium ions. Calcium ions bind to contractile proteins (troponins), initiating the relative movement of thick (myosin) and thin (actin) filaments, resulting in the shortening of sarcomeres^28^. Characterization of the contractile function of an ex vivo heart model can be used to evaluate pathology or drug effects. Therefore, various contraction force detection platforms based on different principles have been developed to assess contractile function. This chapter divides contraction measurements into nonmicrofluidic and microfluidic parts and summarizes recent developments in nonmicrofluidic methods and common microfluidic contraction force measurement platforms.

### Nonmicrofluidic devices

The common conventional nonmicrofluidic methods for detecting cardiomyocyte contractility include the cell drum^29^, two-point force transducers (such as carbon fibers)^30^, atomic force microscopy (AFM)^31^, traction force microscopy (TFM)^32^, and micropillar arrays^33^. The contraction function of the cell drum is characterized by measuring the amplitude and frequency of beating cardiomyocytes. The thin film exhibits little oscillation due to the contraction of cardiomyocytes; however, it causes pressure changes of several Pa in the sealed chamber under the cell drum. Based on these findings, a modified cell drum integrated with a pressure sensor was proposed, and it measured a mechanical stress of 1.62 ± 0.17 μN/mm^2^ and a beating frequency of 3.3 Hz^34^. However, these methods present some issues. For example, AFM may interfere with cells and cannot be used for long-term measurement, and the fluorescent particles in the elastic substrate may be distributed unevenly, thus causing an uneven spatial resolution. Meanwhile, the integration of TFM with other fluorescence-based physiological measurements is limited^35^. Furthermore, drawbacks, such as a low throughput, low dimensionality, and invasiveness, have been identified. Innovation in emerging contraction force sensors in terms of principles, manufacturing processes, materials, etc., is needed to achieve higher technical metrics.

**Fig. 2:**
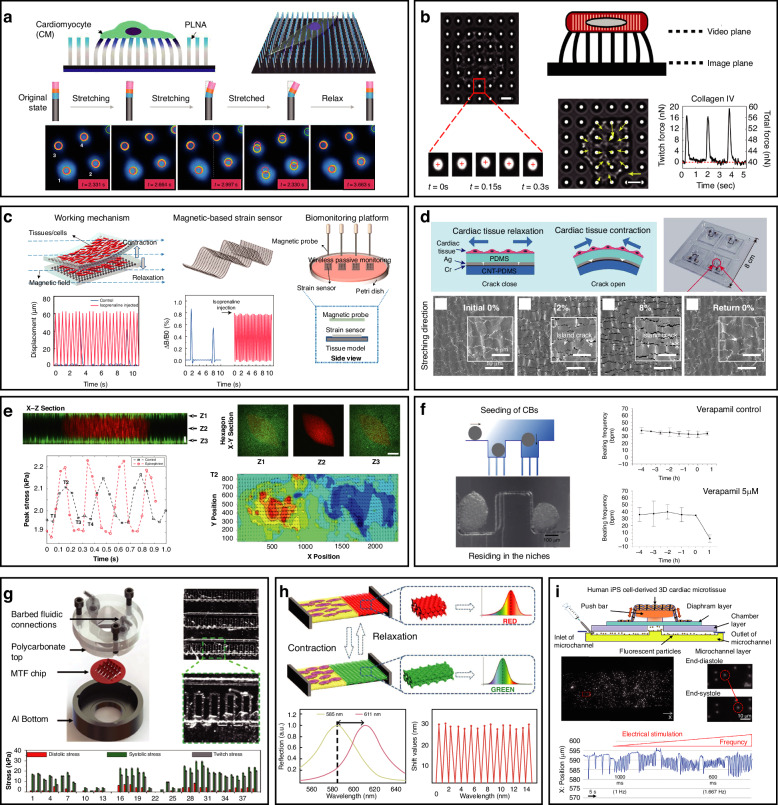
Nonmicrofluidic and microfluidic platforms for measuring cardiomyocyte contractility. **a** Force mapping method based on a “light nanoantenna” array with the piezo-phototronic effect. Reproduced with permission from ref.^41^. **b** Micropost arrays for characterizing the contractile force, velocity, and power produced. Cell contractility was analyzed using microscopy with a high-speed camera. Reproduced with permission from ref.^33^. **c** Magnetic hydrogel-based strain sensors for wireless-passive monitoring. The contraction and relaxation of cardiomyocytes on the hydrogel were evaluated via magnetic field detection. Reproduced with permission from ref.^48^. **d** Working principle and photograph of the Ag/CNT-PDMS sensor. Cardiomyocyte beating results in changes in resistance between nanocracks. Reproduced with permission from ref.^40^. **e** Fluorescence imaging and contractile stress analysis of 3D cardiac tissues. The GelMA hydrogel was used for cell encapsulation, and the PAm hydrogels in the top and bottom layers were used as “stress sensors” for quantifying the contractile stresses generated by the encapsulated cardiomyocytes. Reproduced with permission from ref.^55^. **f** Outline of the microfluidic device with a schematic and picture of the channels used to monitor the beating frequency of cardiac bodies (CBs) via video imaging. Reproduced with permission from ref.^57^. **g** MTF chip and assembly of a microfluidic device for the measurement of contractile stresses. Reproduced with permission from ref.^54^. **h** Working principle and quantitative performance of the graphene hybrid anisotropic structural color film capable of reflecting cardiomyocyte behavior. Reproduced with permission from ref.^61^. **i** A heart-on-a-chip microdevice (HMD) for visualizing the kinetics of cardiac microtissue pulsations by monitoring particle displacement. Reproduced with permission from ref.^70^

Several different principles of sensors for contraction force have been proposed, such as a resistance change^36^, optical analysis methods^37^, and synchronized contraction force and electrophysiological signal measurement^38^. In addition to piezoresistive cantilevers, crack sensors with high sensitivity are used for sensing the contraction force^39,40^. As illustrated in Fig. 2d, the contraction of cardiomyocytes attached to the top layer can cause the opening and closing of cracks in the underlying metal layer (Pt or Ag), and small disturbances in the crack layer can be obtained by measuring changes in resistance. Compared with commercial strain gauges, crack sensors are approximately a thousand times more sensitive. However, the instability of crack sensors is a limitation. For optical analysis, a piezo-phototronic light nanoantenna array in the form of micropillars has been proposed^41^. It utilizes the piezo-phototronic effect to map the traction force generated by cardiomyocytes onto micropillars and reflects it by the photoluminescence (PL) intensity. As shown in Fig. 2a, cardiomyocytes are attached to the InGaN/GaN nanopillars and produce traction force generated by contraction, leading to tiny deformations of the nanopillars. This tiny deformation can induce the redistribution of the piezoelectric potential, thereby modulating the PL emission of the nanopillars. A spatial resolution of 800 nm and a temporal resolution of 333 ms have been reported for traction force mapping. In terms of synchronized contraction force and electrophysiological measurements, a high-throughput drug evaluation platform based on an electromechanical synchronization (EMS) biosensing system has been proposed. This system can establish a database of various cardiac drugs through a heatmap analysis, enabling rapid drug screening^42^. Wang’s team proposed a method using a CMOS multimodal sensor array for cell characterization and drug screening. It consists of multiple pixel groups and parallel signal-mediating blocks. It supports extracellular potential recording, optical detection, charge-balanced biphasic current stimulation, and cellular impedance measurements^43^. In subsequent research, a larger-scale integrated multimodal CMOS cell sensor array was proposed, allowing large-scale, high-throughput and high-content drug screening. The number of pixel groups and signal-mediating blocks increased, and a thermal monitoring function was added^44^.

In terms of sensor manufacturing engineering, 3D printing technology has emerged as a flexible and powerful technique in the advanced manufacturing industry in recent years^45^. K.K. Parker et al. used 3D printing to print and self-assemble a cantilever-shaped cardiac microphysiological device using six functional inks^46^. Strain sensors integrated into cantilevers exhibit changes in resistance following the pulsation of cardiomyocytes. The relationship between the electrical resistance readout and the magnitude of the contraction stress generated by cardiomyocytes was obtained by mathematically modeling the curved cantilever for structural materials and dimensions. Regarding the shortcomings of this method, the sensor exhibited low sensitivity, and issues with material compatibility exist in the 3D printing method. With respect to materials, hydrogels have been discovered, and their properties have gradually improved, making them a promising class of synthetic biomaterials. Hydrogels can be used as an alternative substrate for cardiomyocyte research, providing a matrix stiffness that matches the extracellular matrix (ECM) within the myocardium and better simulates the natural myocardial environment^47^. Furthermore, hydrogels can be modified to achieve enhanced physical properties. By combining a hydrogel with magnetism, a wireless-passive flexible magnetic-based strain sensor was developed, as shown in Fig. 2c^48^. The sensor absorbed the advantages of magnetic devices, such as wireless and passive sensing, while incorporating the high biocompatibility and high-sensitivity properties of the hydrogel. This material exhibited ultrasoft mechanical properties (Young’s modulus of 1.2 kPa), strong magnetic properties (12.74 emu/g), good biocompatibility, and long-term stability (≥ 20 days). Benefiting from the material’s properties, the hydrogel-based strain sensors exhibited a stable monitoring capacity for small strains down to 50 μm.

### Microfluidic platforms

#### Optical measurements

Optical methods have been widely used in contraction force measurements in microfluidic-based platforms due to advantages such as direct visualization and high sensitivity. In the past few years, several optical techniques have been developed for optical detection, including fluorescence, chemiluminescence, and spectroscopic detection methods^49–51^. In the microfluidic device, contraction images or videos of beating cardiomyocytes are captured with a microscope and processed and analyzed with the aid of computer technology to obtain various parameters during the contraction process.

Many early designs of microfluidic chips were used to investigate the contractility of single cardiomyocytes. For example, a microfluidic chip can measure the intracellular Ca^2+^ concentration through fluorescence to monitor changes in contractility and monitor cell shape through optical imaging to confirm the contraction of cardiomyocytes^52^. The unique advantages of this microfluidic approach include the following: the small size of the microchannel makes it easier to sort and retain individual cardiomyocytes than in bulk solution; and the integration of single-cell selection, cell retention, dye loading, chemical stimulation, and fluorescence measurements of intracellular calcium concentrations on a microfluidic chip. K.K. Parker et al. prepared muscle thin films (MTFs) from silicone rubber coated with polydimethylsiloxane (PDMS)^53^. The array cantilever membrane could deflect rhythmically following the contraction and relaxation of the 2D cardiomyocyte layer. The curvature of the cantilever deflection was determined by the displacement observed under a microscope, and the contractile stress was calculated via a formula related to the cantilever curvature. In subsequent work, the authors improved the throughput (10 times) and standardization of the MTF cantilever beams through semiautomated manufacturing technology^54^. In addition, this microfluidic device was embedded with heating elements for temperature control and electrodes for electrical stimulation, as shown in Fig. 2g. It can detect and analyze the mechanical parameters of cardiomyocytes, such as the average peak systolic stress, diastolic stress, and twitch stress. A test drug (isoproterenol, a positive inotropic compound) was applied to the cardiac microtissue. However, a shortcoming of the MTF method is that it only analyzes the parameters of contractility through a post analysis of videos but cannot monitor the contractile forces in real time. The integration of 3D micrograph technology into a 3D cardiac microfluidic device is capable of measuring the contractility of cardiac tissue in real time and in situ, as shown in Fig. 2e^55^. The microchannel-surrounded 3D cardiac tissue was located between two polyacrylamide (GelMA) hydrogels and generated a contraction force to drive the hydrogel layer. The real-time contraction force of cardiac tissue was obtained by analyzing the established mechanical equation and the movement of fluorescent particles embedded in the hydrogel layer. Drug testing with isoproterenol also revealed an increased beating frequency and stress magnitude. This 3D cardiac system with a real-time functional readout could be an attractive technological platform for drug discovery and development. A miniaturized and automated engineered heart tissue (EHT)-based method was proposed to assess the contractile activity of cardiomyocytes and to achieve higher throughput and automation^56^. The systolic and diastolic activities of the spindle-shaped cardiac muscle tissue attached to the silicon pillars were recorded by cameras, and parameters such as the cell contraction frequency, mean force, and contraction and relaxation times were obtained by analyzing shape deformation. This method enables the miniaturization and automation of contractility assessments. These microfluidic methods of measuring contractility enable the direct quantification of the magnitude of the contractile force. However, hydrogel materials are limited by light diffraction, manufacturing accuracy, and GelMA degradation in the presence of cells. These properties result in a lower calculated contractile stress than that previously reported for the heart-on-a-chip. For the EHT method, the manual fabrication of silicon pillars could lead to differences in geometry and elastic properties, which affect the force calculation results.

With the application of more advanced video analysis systems and algorithms for analyzing cardiomyocyte contraction, the measurement of contraction force does not require any auxiliary media or markers, which minimizes errors caused by the manufacturing process to some extent. As shown in Fig. 2f, a microfluidic platform integrated with video microscopy can analyze the beating behavior of cell clusters and obtain contraction parameters through specific algorithms^57^. The iPSC-derived cardiomyocytes were prepared into pellet-shaped cardiac bodies (CBs), and those with diameters between 50 and 200 μm were selected and placed in a microfluidic device for video analysis. The algorithm converts the movement speed of cardiac bodies into a beating frequency by comparing the pixel intensities of two adjacent frames and extracting the information contained in the frames. The contractility of cardiomyocytes was characterized by the beating frequency and exhibited drug effect-related differences in beating frequency in different drug toxicity tests. This approach, with characteristic imaging of cardiomyocytes, provides the opportunity for label-free, noninvasive toxicity studies in 3D microenvironments. The algorithm, which relies solely on the brightness level for cellular oscillation measurements, cannot accurately estimate the cellular oscillations in different regions of the microchip. Moreover, this approach may result in a waveform distortion when the mechanical contraction of cardiomyocytes is characterized. A new algorithm that improves the frame decomposition method for the region of cell pulsation solved the problem of the brightness level measurement^58^. The microfluidic device can assess the mechanical functionality of cells under various electrical field stimuli, and it can depict a heterogeneous distribution map of cell contraction activities within the same spatial domain. Compared with video analysis methods in nonmicrofluidic approaches, these methods possess the advantage of controllable cell dimensions, enabling the contractility of 3D cardiomyocytes of specific sizes to be monitored. However, limitations exist in singular techniques. Due to the small volume of microfluidic devices, the measurements typically rely on direct video analysis, making the integration of the new materials and processes mentioned in the nonmicrofluidic methods above difficult. The microfluidic platforms used for measuring cardiomyocyte contractility are summarized in Table 1.

**Table 1:** Microfluidic-based platforms for characterizing cardiomyocyte contractility

Principle of measurement	Methods of measurement	Quantitative performance	Detected parameters	Dimension of devices	Structure	Drug application	Reference
Mechanical deformation measurement	Calculation of contraction based on the cantilever displacement recorded by microscopy	Average peak systolic stress: 15.4 ± 1.4 kPa, twitch stress: 12.7 ± 1.1 kPa	Systolic stress, twitch stress	Length of the chamber: 11.5×11.5 mm; flow rate: 1 ml/min	2D	Isoproterenol	^54^
	Cantilever vibration recorded by a laser vibrometer	\	Beating frequency, displacement of the cantilever	Dimensions of each cantilever: 3500 μm long, 1500 μm wide, and 16 μm thick	2D	Isoproterenol, quinidine, lidocaine, E-4031, astemizole	^110^
	Contraction force determined by the figure recognition of the contracting muscle strip	Force: 0.1–0.3 mN	Action potential, force, beating frequency, contraction, and relaxation time	Length of the 3D muscle: 8 mm, diameter: 0.2–1.3 mm	3D	Quinidine, doxorubicin, chromanol 293B, erythromycin	^56^
Direct video analysis	Video images analyzed with a designed algorithm	\	Rate of contraction (μm/s), spatial heterogeneity	\	2D	\	^58^
	Beating frequency obtained by the program through the calculation of the pixel intensities	\	Beating frequency	Diameter of media inlets: 0.4 mm; media outlets, inlets for cells and bubble trap: 0.64 mm; flow rate: 0.1 μl/min	3D	Verapamil, quinidine, doxorubicin	^57^
	The variation in the length of the muscle microfibers	\	Relative contraction, beating frequency	Dimensions of the scaffold: 5.5 × 3.5 × 0.75 mm^3^	3D	Doxorubicin	^114^
Fluorescence imaging	Contraction confirmed by fluorescence intensity from an optical imaging system	\	Fluorescence intensity	Diameter of channels: 80.2 μm, depth: 35 μm; diameter of the chamber: 1000 μm, length: 6000 μm	1D	Caffeine	^52^
	Peak systolic stress obtained by calculating the displacement of embedded particles	Order of stress: 1 kPa	Peak stress	Diameter of the inner chamber: 12 mm	3D	Isoproterenol	^55^
	Parameters obtained from the software analyzing the displacement of particles	Detection limit of force: 0.0077 mN	Fluidic output, pressure, force, particle displacement, beating frequency, intracellular calcium oscillations	Diameter of channel: 200 μm	3D	Isoproterenol	^70^
Structural color materials	Color variation due to the beating of cardiomyocytes	Optimal sensing scale: 5–50%	Color wavelength	\	2D	Isoproterenol	^61,62,64^

Direct visualization is another advantage of optical methods in microfluidic techniques. Yuanjin Zhao et al. utilized hydrogels and biological materials to construct structural color films for measuring the contraction forces of cardiomyocytes. By assembling cardiomyocytes on Morpho wings, a visualizable biosensor was developed^59^. The contraction and elongation of cardiomyocytes could drive the flexible wing to deform correspondingly, causing synchronous reflection wavelength shifts and exhibiting color variation. Similarly, they proposed a bioinspired soft robot composed of asymmetric claws, carbon nanotube (CNT)-induced cardiac tissue layers, and structural color indicators^60^. This soft robot powered by cardiomyocytes simulated the crawling behavior of a caterpillar. By integrating these soft robots into a microfluidic chip system with a multitrack structure, they can move along the tracks and exhibit different crawling speeds based on the concentration of isoproterenol on each track, thus reflecting the contraction strength of cardiomyocytes. In addition to their bioinspired structural color design, hydrogels with dynamic structural color have also been developed. The authors proposed a graphene heterogeneous structural color film and microfibers that could reflect the contractility of cardiomyocytes through a color shift, and the film is shown in Fig. 2h^61,62^. The inverse opal structure of SiO_2_ nanoparticles with an ordered arrangement in the hydrogel network endows the film with structural color properties such as photonic bandgap properties and characteristic reflection wavelengths^63^. The contraction and relaxation of cardiomyocytes cause periodic deformation of the structure, resulting in a reversible shift in the structure color. The researchers realized the transformation from micromechanics to macroscopic optics, therefore achieving a microphysiological vision of the heart-on-a-chip. Furthermore, electroconductive and anisotropic structural color hydrogels were fabricated by polymerizing nonclose-packed colloidal arrays on superaligned carbon nanotube sheets (SACNTs)^64^. The excellent mechanical properties, electrical conductivity, and anisotropy of SACNTs make them conducive substrates for biomedical materials.

#### Microdevice integration

Microelectromechanical system (MEMS)-based microfluidic devices (i.e., micromechanical structures, microelectronic devices, and microsensors) have also gained popularity in cardiomyocyte contractility measurements because of their miniaturization, multifunctionality, and high degree of integration^65^. Si is a commonly used piezoresistive cantilever material with the advantages of high sensitivity and stable physical and chemical properties. A sensor for measuring the contraction force of cardiomyocytes via MEMS piezoresistive cantilevers was proposed^66^. It features a high frequency (tens of kHz) and a high sensing resolution (less than 0.1 nN). Lee et al. proposed an SU-8 cantilever with an integrated single-crystal silicon strain sensor, whose main feature is high sensitivity^67^. The 2-μm-thick silicon strain sensor could produce a resistance variation for small deformations and detect a small contractile force of less than 0.02 μN. This device demonstrated the potential for fabricating high-throughput cantilever arrays and their applications in microfluidic platforms. Polyimide (PI) can also serve as a material for cantilevers^68^. The PI cantilever is highly heat resistant while retaining high biocompatibility, allowing for metal deposition and sterilization. The contraction force generated by cardiomyocytes is characterized by cantilever displacement measured using a laser vibrometer sensor. In a cantilever design with a similar principle, the deflection of the cantilever tip is determined via the silicon cantilever, the photodetector, and the dimensions and parameters of the components. The size of the cantilever, the physical properties of the single-crystal silicon, and the thickness of the cardiomyocyte layer (21 ± 1 mm measured by confocal laser microscopy) were subsequently substituted into the modified version of the Stoney equation. Cantilever deflection can be converted into the contraction force generated by the cardiomyocyte layer^69^. By combining human iPSC-derived cardiac tissue and MEMS, a heart-on-a-chip microdevice (HMD) was proposed to evaluate the contractile function of cardiac tissues, as shown in Fig. 2i^70^. Contractile function was assessed via a video-based system, and the change in cardiomyocyte beating after electrical stimulation was demonstrated. The device could also visualize the dynamics of heart microtissue beating by monitoring the displacement of particles, which allowed the quantification of physiological parameters, including fluid output, pressure, and force. The change in particle displacement after the application of isoproterenol led to a change in the fluctuation rate, indicating the accurate prediction of the pharmacological response to inotropic agents.

## Monitoring electrophysiological signals

### Electrophysiological monitoring using nonmicrofluidic devices

The patch clamp technique is the gold standard for single-cell-level electrophysiological monitoring and can record and manipulate the currents that flow through individual ion channels or the entire cell membrane^71^. It can analyze parameters such as the action potential, I-type calcium current, and basal inward rectifier current. Specifically, after a high-resistance seal is formed, the cell membrane near the tip of the microelectrode is ruptured via slight negative pressure (suction) or a brief electrical pulse. This step allows the internal solution of the electrode to mix with the intracellular fluid, enabling the recording of the electrical activity of the entire cell^72^. A modified patch clamp dispensed the cell suspension toward the tip of glass micropipettes to form a gigaseal. The stable seals and access resistance guarantee a high recording quality. However, the patch clamp technique requires considerable experience and skill and is time-consuming. In addition, it can harm cells during the experimental process.

Optical methods are also used for cell electrophysiology research. Optogenetics is a technique that uses light-sensitive proteins and genetic engineering to control specific cellular activities^73^. The optogenetic approach focuses on the action potential waveform generated by a monolayer of human cardiomyocytes, thus assessing the contributions of several ion channels to the overall effect of a drug^74^. A method combining optogenetic stimulation and optical imaging was used to detect hiPSC-CM electrophysiology. The system integrated optogenetic stimulation with the simultaneous recording of membrane voltage and intracellular calcium dynamics using a single photodetector, demonstrating high spatiotemporal resolution in the context of cardiotoxicity testing^75^. Moreover, optical pacing via infrared light and fluorescence mapping was incorporated to quantify cardiac electrophysiological parameters in a standard 96-well plate^76^. The method can record the action potentials and Ca^2+^ transients of cardiomyocytes, analyzing the conduction velocity and action potential duration in a single well. Other commonly used methods include microelectrode arrays (MEAs) and light addressable potentiometric sensors (LAPSs)^77^. MEAs, which are capable of noninvasive and multichannel recording, have become widely used tools in electrophysiological recording.

### Electrophysiological monitoring using microfluidic devices

#### Monitoring action potentials

The action potential (AP), which is a membrane potential-driven waveform that affects ion channels and leads to the occurrence of Ca^2+^ transport, is the initial event of cardiac ECC coupling. The AP is also responsible for the propagation of excitation information between cardiomyocytes, allowing the heart to function as a functional syncytium (both electrically and mechanically)^71^. A patch clamp can be used to record the ion channel activities of the cell membrane and obtain information about action potential occurrence. Patch clamps have continuously progressed, achieving automation, high throughput, and the analysis of multiple electrophysiological parameters^78^. Compared with manual patch clamp methods, which require experimental skills and experience, this system has improved operational convenience and data repeatability. Moreover, it can be directly applied to cell phenotyping and drug screening. In recent years, other methods have also emerged, including microelectrode arrays (MEAs)^79,80^, CMOS nanoelectrode arrays (CNEAs)^81^, calcium imaging^82,83^, voltage-sensing optical (VSO) platforms^84^, and impedance spectroscopy^85^. Copper et al. proposed a microfluidic method for the electrical stimulation and recording of evoked action potentials from single cardiomyocytes^86^. The device divided the cell’s outer space into two distinct microfluidic pools, the contents of which were controlled and rapidly switched between solutions via a pair of concentric automated pipettes. Chemical stimulation was achieved by locally applying K^+^ and caffeine, and action potentials were recorded through microelectrodes in the pipette and Ca^2+^ transient measurements. In other relevant studies, this team explored cell-to-cell signaling between longitudinally linked primary cardiomyocytes^87^. One important reason for choosing pairs of cardiomyocytes is that individual cardiomyocytes are mechanically and electrically active, and monitoring synchronized activation via optical and electrical recording is easy due to intercellular coupling. This study explored the electrical and mechanical coupling between two cardiomyocytes and monitored the propagation of Ca^2+^ waves and the effects of a drug (caffeine). This measurement was achieved by optical recordings, including fluorescence and cell contraction.

**Fig. 3:**
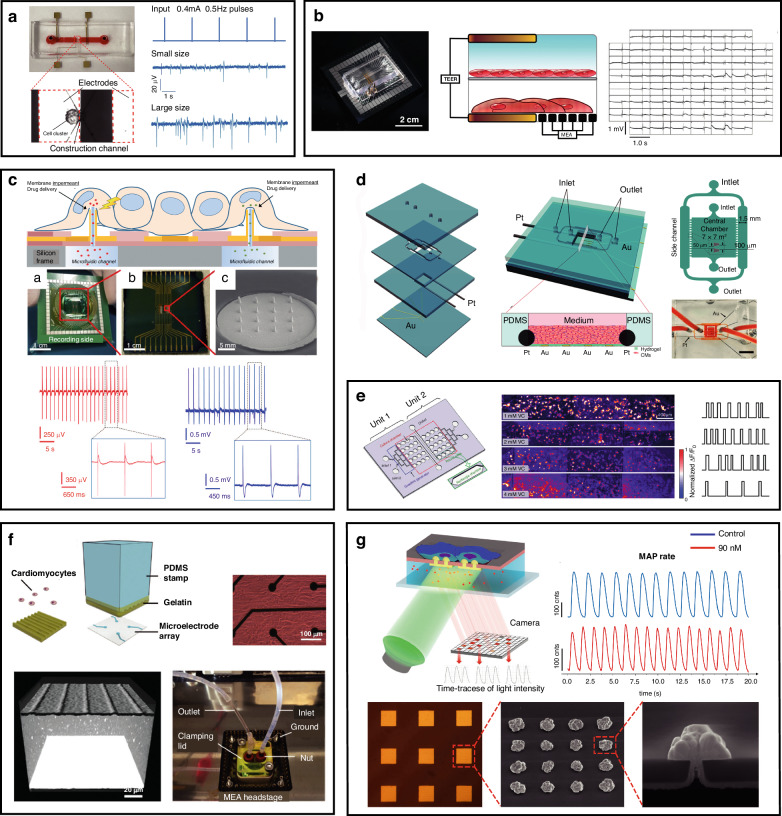
Microfluidic platforms for monitoring electrophysiological signals. **a** Microfluidic device integrated with electrical stimulation for measuring field potentials and distinguishing excitable cells from electrically non-excitable cells. Reproduced with permission from ref.^101^. **b** Photograph and schematic of the organ-on-a-chip integrating both MEAs and TEER measurements. Dynamic detection of vascular permeability and cardiac function under the inflammatory stimulus tumor necrosis factor-alpha (TNF-α) or the cardiac-targeting drug isoproterenol. Reproduced with permission from ref.^98^. **c** Multielectrode array decorated with 3D hollow nanotubes integrated with microfluidic channels for electrical recording and drug delivery. Reproduced with permission from ref.^93^. **d** Heart-on-a-chip for the long-term dynamic culture of cardiomyocytes and field potential recording with Au electrodes. Reproduced with permission from ref.^102^. **e** High-content drug screening (10 types of drugs, each with 5 concentrations to be assayed simultaneously) using high-resolution Ca^2+^ imaging. Reproduced with permission from ref.^90^. **f** Laminar cardiac tissues formed through topographical cues and integration with commercial MEAs in a microfluidic device. Reproduced with permission from ref.^97^. **g** Microfluidic device for action potential recording, where cell ionic currents are transduced into mirror charges. It detects the effects of nifedipine recorded as MAPs (mirror action potentials). Reproduced with permission from ref.^91^

In addition to electrophysiological recordings of primary cardiomyocytes, recordings of the electrophysiological properties of action potentials (APs) and ionic currents in hiPSC-derived cardiomyocytes (hiPSC-CMs) have been performed^88^. Electrophysiological recordings of higher-dimensional (3D) tissues have been reported. A three-dimensional nanoelectronic array simulating a tissue scaffold comprising 64 addressable devices with subcellular dimensions and submillisecond temporal resolution was proposed^89^. Real-time extracellular action potential (AP) recordings reveal a quantitative map of AP propagation in 3D cardiac tissue, allowing in situ tracking of the evolving topology of 3D conduction pathways in developing cardiac tissue. Furthermore, this study provides further evidence that synchronous multipoint stimulation and mapping can control the frequency and direction of AP propagation. The device, which combines microfluidics and video analysis, enables high-content screening (of multiple drugs and concentrations)^90^. As shown in Fig. 3e, this work demonstrates action potential-induced Ca^2+^ influx and reveals the spatiotemporal pattern of cardiomyocyte Ca^2+^ imaging, possibly providing an advanced alternative to plate-based approaches. The mirror charge concept from classical electrodynamics was introduced to measure the action potentials of cardiomyocytes, as shown in Fig. 3g^91^. The device converts cellular ionic currents into mirror charges in a microfluidic chamber. The fluxes of fluorophores caused by cell ionic transmembrane currents are transduced into a light signal that can be detected and recorded with a standard optical camera. The device enables high-quality and noninvasive electrophysiological measurements of cardiomyocytes.

For high-throughput and long-term dynamic culture, Wei et al. proposed a microfluidic-driven uninterrupted perfusion system (HT-μUPS)^92^. HT-μUPS microplates can be integrated with commercially available microplates, and acute/chronic all-optical electrophysiology studies can be conducted via microscopy or long-term cell/tissue culture in a standard incubator. This scalable perfusion system can help with in vitro pharmacokinetic and pharmacodynamic studies. Cerea et al. proposed a multifunctional MEA to achieve spatial resolution in the handling of single-cell drug delivery, as shown in Fig. 3c^93^. Each microelectrode was decorated with 3D hollow gold nanotubes. In essence, by exploiting the favorable geometric characteristics of 3D hollow nanoelectrodes, electroporation could achieve direct access to the cell interior with high spatial localization. The proposed MEA could realize action potential recording and intracellular recording, as well as selective intracellular drug delivery through electroporation.

#### Recording field potentials

Microfluidic devices are capable of recording the extracellular field potential of a single cell in a noninvasive and high-throughput manner. Cooper et al. proposed a microfluidic device that separates the outer space around cardiomyocytes into three compartments^94^. Individual cardiomyocytes were aligned across a microchannel and bridged two microfluidic channels located on the electrically insulating gap. During the process of action potential induction by electrical stimulation, the extracellular potential and current of single cells were recorded, as were the sarcomere length and Ca^2+^ transients at different stimulation rates. This insulating gap also enables the independent manipulation of the extracellular environment at the cell end, creating gradients of chemicals with the aim of detecting effects on cardiomyocyte behavior. The authors also proposed a microfluidic system based on five independently addressable microelectrodes^95^. This electrochemical microbial sensor can measure the amount of lactate produced by cardiomyocytes, as well as the extracellular pH and Ca^2+^ transients. The subjects of the above studies are single cardiomyocytes, which have the capacity to conduct simultaneous electrochemical and optical detection of metabolites in a single cardiomyocyte. However, such methods fail to capture the electrical coupling of cardiomyocyte populations and long-term dynamic monitoring.

MEA is a common 2D cardiomyocyte electrophysiological monitoring method^96^. Parameters of field potentials (such as amplitude, duration, and discharge rate) can be analyzed via MEA recordings. The noninvasive nature of MEAs makes them a preferred tool for recording long-term field potential signals^35^. In particular, MEAs have been combined with microfluidics to achieve electrophysiological measurements in various applied settings. The groove-shaped topography constructed on soft gelatin can induce the regularly arranged growth of cardiomyocytes to promote maturation. A microfluidic device integrated with this method and a commercial MEA successfully recorded electrophysiological signals, and drug testing of isoproterenol using hiPSC-CMs was performed, as shown in Fig. 3f^97^. In the endothelialized heart model, transepithelial electrical resistance (TEER) is used to characterize endothelial cell barrier function, in addition to an MEA for field potential measurement. As shown in Fig. 3b, the TEER-MEA chip integrated the MEA and TEER electrodes to achieve dynamic detection of vascular permeability and cardiac function^98^. The experimental results of TEER and MEA revealed that the chip could simultaneously detect dynamic alterations in vascular permeability and cardiac function under the conditions of the inflammatory stimulus tumor necrosis factor-alpha (TNF-α) or the cardiac-targeting drug isoproterenol. Thus, this organ-on-a-chip with an integrated sensing capability may prove useful for real-time assessments of biological functions, as well as the response to therapeutics. In addition, biosensor integrating MEAs and interdigital electrodes (IDEs) can be used to measure cell viability and electrophysiological activity simultaneously^99^. Compared with platforms that record the field potential only, this chip enables the measurement of beating amplitudes and intervals of cardiomyocytes. The microfluidic platforms used for monitoring electrophysiological signals are summarized in Table 2.

**Table 2:** Microfluidic-based platforms for monitoring cardiomyocyte electrophysiological signals

Type of electrophysiological signal	Method of measurement	Quantitative performance	Detected parameters	Dimension of devices	Structure	Stimulation	Drug application	Reference
Action potential	Electrochemical monitoring	\	Ca^2+^ transients, intracellular pH, cell shortening	15 chambers; width of chambers: 50 mm, length: 250 mm	1D	ES	\	^64^
	Cellular ionic currents are transduced into mirror charges	\	Fluorescence intensity		2D	\	Nifedipine	^91^
	All-optic electrophysiology	\	Field potentials, calcium transients	Width of channels: 1.5 mm, height: 0.5 mm; diameter of inlets and outlets: 1.5 mm	2D	ES	\	^92^
	Multielectrode array and hollow nanotubes integrated on microfluidic channels	\	Field potentials, intracellular-like action potential	Diameter of nanotubes: 600 nm, height: 1.3 μm; flow rate: 100 μl/min	2D	Electroporation	Intracellular delivery of calcein-AM, propidium iodide (PrhD-1)	^93^
	Spatiotemporal patterns of Ca^2+^ imaging	Calcium wave propagation	Fluorescent intensity ΔF/F_0_	Diameter of the cell culture well: 360 μm; field of calcium signal imaging: 1.0 × 1.1 cm	2D	K^+^ solution	Butyrate, deoxycholate, citric acid, vitamins, etc.	^90^
Field potential	Commercial MEA integrated with microfluidic platforms	\	Beating rate, field potential, conduction velocity	Flow rate: 60 μl/h	2D	\	Isoproterenol	^97^
	Au electrode for recording combined with an analysis of motion tracking of cardiomyocytes	Contraction speed (stimulated): 40.5 μm/s (1 week), 32.4 μm/s (4 weeks)	Beating speed, beating rate, field potential	Diameter of inlets and outlets: 1.6 mm; length of chamber: 7 × 7 mm; flow rate: 1.5 μl/min	2D	ES	Isoproterenol, verapamil	^102^

#### Electrical stimulation

Electrical stimulation (ES) plays an important role in regulating cell behavior and is widely applied in electrophysiological research. A microfluidic device designed for single-cell electrical stimulation and recording extracellular field potentials allows for the continuous application of ES and monitoring of field potentials within the microfluidic channel^100^. In addition, a microfluidic device capable of rapidly applying various electrical stimulation signals has been developed, enabling noninvasive discrimination between electrically excitable and non-excitable cells while recording field potentials, as shown in Fig. 3a^101^. Zhang et al. developed a heart-on-a-chip device utilizing Au electrodes for extracellular field potential recording and platinum electrodes for electrical stimulation, as shown in Fig. 3d^102^. hiPSC-derived cardiomyocytes were seeded on in situ gelatin hydrogels prepared in the chamber and subsequently divided into control and stimulation groups. After a 4-week culture period, the stimulation group exhibited a more mature phenotype characterized by stable electrical activity rhythms and increased expression of cardiac-specific proteins. After treatment with verapamil, cardiomyocytes only exhibited normal electrophysiological responses to isoproterenol when subjected to electrical stimulation, indicating the reliability of this heart-on-a-chip platform for application in drug efficacy testing and cardiotoxicity screening.

Electrical stimulation for the regulation of cell behavior has been used in various biomedical studies. For example, ES has been shown to influence cell adhesion, migration, proliferation, and secretion of the extracellular matrix^103^; embryoid bodies of hiPSCs promote the differentiation of cardiomyocytes after exposure to acute ES^104^. Furthermore, ES of iPSC-CMs in three-dimensional culture can enhance the maturation and functional assembly of cardiomyocytes into engineered contractile heart tissues^105^. ES can also help identify cells of the desired phenotype from the heterogeneous population generated from stem cell differentiation. For example, Myers et al. proposed a nongenetic, label-free cytometric approach based on electrophysiological stimulation responses^106^. The ES microfluidic system is capable of distinguishing undifferentiated iPSCs from iPSC-derived cardiomyocyte clusters via electrical stimulation and recording extracellular field potential signals of suspended cells in liquid flow.

## Applications of electromechanical measurements in microfluidic platforms for cardiomyocytes

### Drug screening

The adverse reactions of the heart to drugs represent a primary risk in drug development^107^. This risk can be attributed to the fact that cardiotoxicity is the most frequent and severe adverse reaction in the late stage of clinical drug development^108^. When cultured in vitro, cardiomyocytes can exhibit regular contractile behavior. The resulting contraction force can be used as a readout to examine tissue responses to various environmental cues, including small chemical molecules and drugs, which serve as relevant indicators in toxicology research^109^. A high-throughput drug screening platform has been proposed to achieve high-throughput screening of diverse drugs^110^. Cardiac toxicity levels were assessed by measuring changes in the contraction force of cardiomyocytes. This device consisted of 48 wells with a total of 192 SU-8 cantilevers. The presence of four interlocking cantilever structures in each well enhanced the reliability of the data, and the microgroove pattern reinforced the anisotropic arrangement of cardiomyocytes. In this study, isoproterenol, verapamil, quinidine, E-4031, and lidocaine were used to validate the high-throughput drug screening capability.

**Fig. 4:**
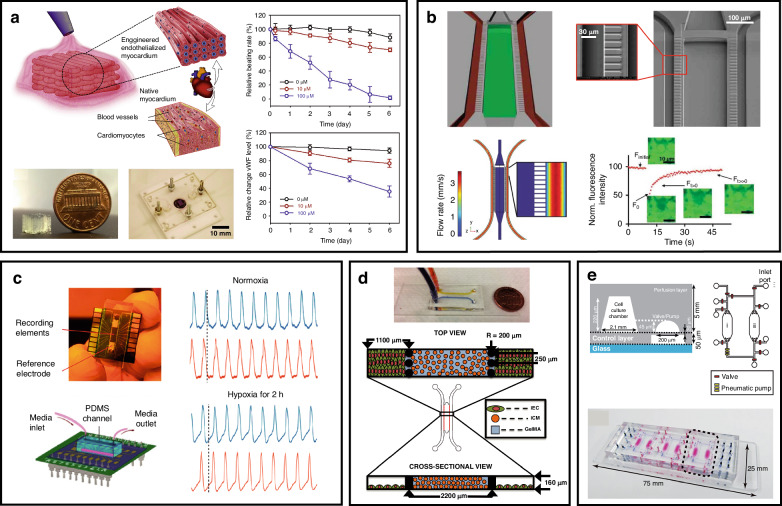
Microfluidics for drug screening and disease modeling. **a** Microfibrous scaffolds for evaluating cardiovascular toxicity in the engineered endothelialized myocardium. Reproduced with permission from ref.^114^. **b** Cardiac microphysiological system containing nutrient channels and cell channels. Reproduced with permission from ref.^112^. **c** Heart-on-a-chip for studying the effects of acute hypoxia on cardiac function. Reproduced with permission from ref.^129^. **d** Microfluidic myocardium-on-a-chip for CMs and ECs cocultured with capillary-like channels. Reproduced with permission from ref.^113^. **e** Microfluidic device integrated with CMs and liver cancer cells to simulate the side effects of the anticancer drug doxorubicin (DXR). Reproduced with permission from ref.^115^

Microfluidics has unique advantages for generating microtissues, with the ability to integrate perfused and engineered tissues, which can enhance the transport and diffusion of nutrients. Specifically, it can be described as allowing rapid diffusion, such as mass and heat transfer; accurately simulating physiological conditions through cell-based analyses; and ensuring a continuous supply of nutrients and oxygen^111^. Mathur et al. developed a microphysiological system (MPS) with two primary functions: (i) using 3D confinement with biomimetic dimensions to facilitate self-organization of hiPSC-CMs into an aligned 3D tissue and (ii) mimicking the shear flow protection of the endothelial barrier^112^. As shown in Fig. 4b, the MPS consists of a central cell chamber, two adjacent media channels, and an array of connecting microchannels. The media channels (widths of 30–40 μm) emulate the vascular system, enabling the precise and predictably calculable delivery of nutrients and drugs into the microfluidic chamber. In pharmacological testing, beating frequency is employed as a metric, and four drugs representing different drug categories are utilized: isoproterenol (β-adrenergic agonist), E-4031 (hERG blocker), verapamil (multi-ion channel blocker) and metoprolol (β-adrenergic antagonist). The results of drug testing revealed that the IC50 value of verapamil in the microscopic system (MPS) is greater than that of embryonic body-derived cardiomyocytes (CMs), indicating that MPS has greater tissue maturity and development. As illustrated in Fig. 4d, a myocardium-on-a-chip (MOC) model was proposed to simulate the human myocardium and microvasculature in natural cardiac tissues in vitro^113^. A 3D spatial control was used for the coculture of hiPSC-differentiated cardiomyocytes and endothelial cells. Cardiomyocytes were cultured in the central channel, with functionality characterized by calcium imaging and MEA recordings. Endothelial cells were cultured in capillary-like side channels, with shear stress simulated by different perfusion rates. The expression of endothelial surface markers and adhesion proteins revealed the functionality of mature endothelial cells. Characterization of the viability of the MOC revealed a uniform distribution of viable cells throughout the central channel, and cell viability remained consistent throughout the culture duration. The device can be used to scrutinize procedures and drug treatments for CVD before progressing to more costly animal models and, in turn, can increase the success rate of drug development and procedures.

A 3D bioprinting strategy was applied to create a scaffold for mimicking the hierarchical structure of the native myocardium and integrating the vasculature^114^. As shown in Fig. 4a, this approach directly bioprinted a composite bioink containing endothelial cells onto a microfiber hydrogel scaffold, thus generating a more physiologically relevant organoid resembling the human cardiovascular system. Through the use of the composite bioink, the endothelial cells directly bioprinted within the microfibrous hydrogel scaffolds gradually migrated toward the peripheries of the microfibers to form a confluent endothelial layer. The resulting organoids were embedded into a perfusable microfluidic device, and an assessment of the vascular toxicity of the anticancer drug doxorubicin (DXR) was conducted. In the application of the same drug, a microfluidic device integrating a heart/cancer chip was designed to replicate the side effects of doxorubicin (DXR)^115^. As illustrated in Fig. 4e, this device comprises three sets of artificial blood circulation loops, allowing the cultivation of individual cells from different tissues. Pneumatic valves and peristaltic pumps are utilized to control fluid flow, enabling precise fluid dynamics that can simulate the side effects of DXR on cardiomyocytes.

### Disease modeling

Microfluidic platforms also allow the study of genetic disorders under physiologically relevant conditions because of the compatibility between small sample sizes and primary or patient-derived cells. An analysis of pathogenesis is considered pivotal in cardiovascular disease studies because it not only provides physiological and pathological information for the foundational research of cardiovascular diseases but also provides a research basis for diagnosis and treatment^24^. For example, the methods for genetic cardiovascular disease modeling involve inducing pathology by introducing gene mutations to modify hiPSC-CMs. A heart-on-a-chip model was proposed to elucidate the pathophysiological basis of Barth syndrome (BTHS) cardiomyopathy^116^. Using BTHS iPSC-derived cardiomyocytes (iPSC-CMs), metabolic, structural, and functional abnormalities associated with TAZ mutations were identified. New insights into the pathogenesis and treatment strategies for Barth syndrome was proposed through characterization of the sarcomere phenotype and metabolic and functional analyses of cardiac tissue in the assembled heart-on-a-chip.

In addition to modeling genetic cardiovascular diseases, microfluidics can replicate the inherent features of the cardiovascular system and handle small-volume liquids (10^−9^–10^−18^ L)^9^. The intricate structures in the microvascular network and control of fluid flow in microchannels can be simulated to achieve biomimicry of cardiovascular hemodynamics^117^. As a method to investigate the pathology of arterial and venous thrombosis in cardiovascular diseases, microfluidic systems have been utilized to create thrombi rich in platelets. Optical microscopy and histology were employed to measure thrombi under different shear rates^118^. By distinguishing from the physical perspective of the pathogenesis of thrombus formation, Abhishek et al. investigated the physiology of thrombi by focusing on biological factors such as vascular endothelial cells^119^. They explored whether a microfluidic device lined by a chemically preserved (fixed) human endothelium will retain its ability to support thrombus formation and platelet adhesion when human whole blood flows through the channel at an arterial shear rate. The results demonstrated the potential clinical value of this device by showing that thrombus formation and platelet function could be measured within minutes using a small volume (0.5 mL) of whole blood collected from subjects receiving antiplatelet medications.

Atherosclerosis is a common form of cardiovascular disease. Endothelial cells are critical subjects in the study of atherosclerosis and are the primary regulators of vascular homeostasis. Because they serve as the interface between blood and tissues, endothelial cells are most susceptible to changes in blood components and hemodynamics, playing a pivotal role in the development mechanism^120^. In arteries, endothelial cells (ECs) are constantly subjected to two types of hemodynamic force, fluid shear stress (FSS) and cyclic stretch (CS), both of which result from blood flow and blood pressure^121^. A physiological level of mechanical force is necessary for the development and maintenance of the normal structure and function of vessels. Zheng et al. proposed a microfluidic-based disease model for early-stage atherosclerosis (AS). This microfluidic device utilizes pneumatic mechanical stimulation to simulate several conditions of the vascular hemodynamic environment, reproducing the impacts of abnormal FSS and CS on endothelial dysfunction^122^. Based on the physiological range of FSS in human arteries (1.5–7.0 Pa over the cardiac cycle), 5.07 Pa was selected as the physiological condition, and a lower value of 1.16 Pa was selected as the pathophysiological FSS. Compared with cells grown in static Petri dishes, the results indicate that the cells in the early-stage AS model are more sensitive to biochemical stimulation. Furthermore, the study also assessed the efficacy of the anti-AS drug probucol.

Inflammation, which aids the body in preventing microorganism invasion, has been reported to be detrimental to atherosclerosis. The number of heart attacks caused by inflammation surpasses that caused by cancer^123^. Most heart-on-a-chips that coculture endothelial cells with cardiomyocytes have been used to assess endothelial barrier function under inflammatory conditions to faithfully replicate the atherosclerosis model. For example, endothelialized heart-on-a-chips were created by integrating MEAs and electrodes for epithelial resistance measurements into a microfluidic chip^98^. In addition to applying MEAs to measure the field potential of cardiomyocytes, this chip can simultaneously measure tissue barrier resistance through TEER, which enables rapid, label-free, and highly sensitive measurements of barrier integrity and permeability. Vascular permeability and cardiac function can be dynamically monitored under inflammatory stimuli, such as tumor necrosis factor or isoproterenol stimulation. The inflammatory response of endothelial cells can lead to increased vascular permeability through gap formation. Thomas et al. compared changes in vascular permeability during inflammation between the biomimetic blood vessel (BBV) microfluidic model and the conventional Transwell insert model along with the permeation characteristics of different tracer molecules through thrombin-induced endothelial cell layers in both models^124^. The results revealed similar permeabilities, but the transport of large molecules in the BBV model was more suitable than that in the static Transwell insert model. A perfusable vascularized microchannel capable of creating various geometric shapes through an extracellular matrix hydrogel patterning method has been proposed^125^. Changes in endothelial barrier permeability during inflammation and neutrophil transmigration across the endothelium were investigated using this device, creating a biomimetic 3D endothelial-smooth muscle cell vascular model. The results indicated a significant reduction in barrier permeability in the coculture model during inflammation and narrowing of collagen structure vascular microchannels in atherosclerosis. These findings illustrate the unique advantages of microfluidic technology in mimicking endothelial barrier function and vascular permeability.

Acute myocardial infarction (AMI) is a major disease that contributes to mortality and disability worldwide and is clinically characterized by myocardial necrosis and acute myocardial ischemia^126^. AMI is caused by the interruption of the blood supply to a part of the heart, and subsequent hypoxia may result in myocardial tissue damage or even death if the condition is not treated promptly^127^. Using a micropillar array to mimic tissue interfaces and hypoxia to establish an oxygen concentration gradient, an AMI model was developed on a microfluidic chip^128^. The chip induced hypoxia via the chemical solution of FCCP, which caused cell hypoxia by disrupting the mitochondrial proton gradient and preventing ATP synthesis. Immunofluorescence was employed to characterize apoptosis of cardiomyocytes through staining for sarcomeres and relevant protein and gene expression levels. Fluorescence images revealed a reduction in the mitochondrial membrane potential and increased expression of the apoptosis gene Caspase-3 in hypoxic regions. In addition to staining for the characterization of cellular viability, electrophysiological signals can be used to study the effects of acute hypoxia on cardiac function, as illustrated in Fig. 4c^129^. The designed channels can rapidly adjust medium oxygenation, simulating the mechanisms induced by temporary coronary artery occlusion. For electrophysiological signal recording, the Pt nanopillars temporarily entered the cytosol after electroporation, yielding action potential (AP)-like readouts. The results revealed that APs narrowed during hypoxia, which is consistent with the proposed mechanisms by which oxygen deficits activate ATP-dependent K^+^ channels that promote membrane repolarization. In studies designed to understand the underlying molecular mechanism, microfluidic single-cell profiling indicated that iPSC-CMs could release significant levels of proangiogenic and antiapoptotic factors in the ischemic microenvironment, which can be demonstrated by laser capture microdissection of host myocardium, and in vitro ischemia stimulation^130^.

Heart failure (HF) is a common complication of myocardial infarction and is caused primarily by cardiac hypertrophy induced by an excessive volume load^131^. A microfluidic device was used to investigate the impact of hydraulic pressure on cardiomyocytes, allowing the application of high hydraulic pressure while minimizing shear stress and its influence^132^. A pressure of 170 mmHg resulted in an increased cell area and the expression of atrial natriuretic peptide (ANP), which serves as a metric for assessing the response of cardiomyocytes to hemodynamic stress. This study tested the effects of a focal adhesion kinase (FAK) inhibitor on these pressure-induced responses, and the results suggested that FAK was involved in pressure-induced signaling pathways in cardiomyocytes. This mechanical‒chemical antagonism offers a potential therapeutic strategy for hypertension-induced cardiac hypertrophy. An air-driven microfluidic platform for high-throughput studies of cardiac hypertrophy allows for the repetitive (up to one hundred thousand times) and robust (over several weeks) manipulation of cardiac microtissues. This model faithfully simulates cardiac hypertrophy induced by volume overload and can test thousands of loading conditions^133^.

With the advantages of dynamic perfusion and control of the microvolume, microfluidics has become a useful tool for the in vitro simulation of cardiovascular diseases, facilitating studies of disease pathogenesis and even examinations of treatment strategies. In the future, it will possibly evolve into a robust research tool for various diseases.

## Design strategies

**Fig. 5:**
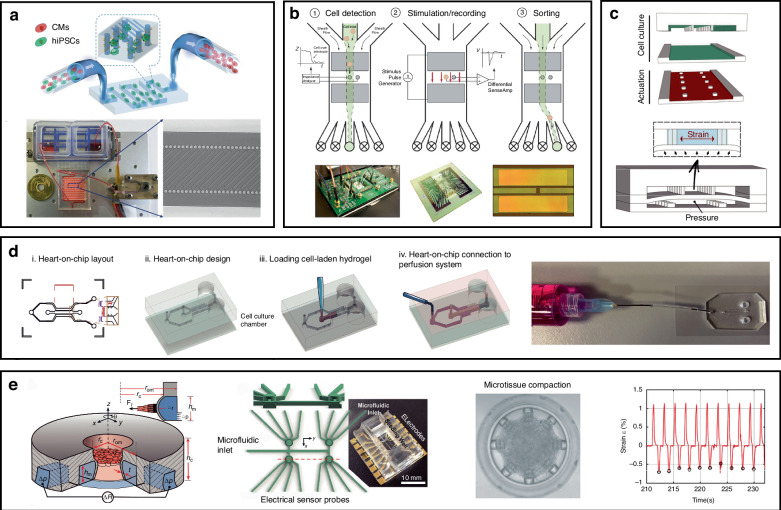
Microfluidic platform design strategies for monitoring the physiological signals of cardiomyocytes. **a** Microfluidic device for hiPSC-CM purification comprising a peristaltic pump, cell suspension reservoirs, and a serpentine channel. Reproduced with permission from ref.^137^. **b** Microfluidic device for electrophysiological cell sorting in response to stimuli. Reproduced with permission from ref.^138^. **c** Heart-on-a-chip providing mechanical and biochemical costimulation. Reproduced with permission from ref.^143^. **d** Perfusable system connected with a heart-on-a-chip for measuring myocardial‒microvascular interactions. Reproduced with permission from ref.^145^. **e** Microfluidic platform fabricated by DLW lithography and soft lithography for monitoring oscillatory forces generated by microtissues under a prescribed mechanical loading and pacing. Reproduced with permission from ref.^146^

Microfluidic-based platforms can be used for monitoring cardiomyocyte contractility and electromechanical activity, drug screening, and CVD modeling with various design strategies. Therefore, this chapter aims to summarize the design strategies, with the goal of enriching the relevant physiological studies on cardiomyocytes. In the process of extracting neonatal rat cardiomyocytes, an essential step is to separate the cardiomyocytes from the cell suspension. Leveraging the physical characteristics of cells, a size-based microfluidic device was designed as a diffusive filter for cell enrichment^134^. Similarly, the principle of deterministic lateral displacement was used to design a microfluidic device for enriching cardiomyocytes^135^. At the single-cell scale, Espulgar et al. utilized centrifugal microfluidic technology to capture individual cardiomyocytes from primary cultures and control the cell separation distance^136^. Similar to the collection of primary cardiomyocytes, multiple cell types also exist in iPSC-derived populations. Cardiomyocytes must be separated and purified to obtain a pure population of these cells. A microfluidic device integrated with a surface-functionalized fishnet-like structure was designed for purifying hiPSC-derived cardiomyocytes^137^. As shown in Fig. 5a, the cells in the solution passed through the fishnet-like structure, with the selective retention of the chosen cell population. The process does not compromise cell viability, with a capture rate over 80% for hiPSCs. In addition to physical methods of purification, electrophysiological measurements can be employed to sort hiPSC-CMs and undifferentiated embryoid bodies, as illustrated in Fig. 5b^138^. This device uses the recording of the characteristic field potential to sort cells, and it can mitigate artifacts generated in the recording process through the application of the geometric structure of differential electrodes and an artifact suppression algorithm.

Although the differentiation of hiPSCs into contracting CMs is well established, their maturation into adult-equivalent cells and their formulation into functional adult-like tissue remain unmet challenges^139^. The mechanical load, matrix stiffness, and electrical stimulation applied to cardiomyocytes can influence the cell alignment, contraction frequency, and contraction strength, manifesting as distinct maturity and functional phenotypes^140^. Sethu’s group integrated mechanically loaded cardiomyocytes with fluid flow and circulation networks to simulate cardiac loading conditions and develop a microfluidic cardiac circulation model^141^. The thin film in the device is used for cell culture and can undergo mechanical stretching through pneumatic actuation. Fluid in the circulation network constantly flows to generate varying shear stress levels, allowing for a dynamic adjustment of the preload and afterload by modulating the pump flow rates. The simulation of in vivo mechanical conditions, including the circulating fluid flow, chamber pressure, and other potential strains, has shown that early mechanical stimulation of cardiac tissue is necessary for protein synthesis of the calcium-handling components required for tissue proliferation and contraction^142^. Similarly, the heart-on-a-chip method developed by Marsano et al. uses a pneumatic driving system to apply uniform uniaxial cyclic strain to a 3D cell structure, as depicted in Fig. 5c. The application of a dynamic load achieved enhanced cardiac tissue differentiation, as well as electrical and mechanical coupling. This approach yields mature and highly functional cardiac tissue^143^. Moreover, maturation is affected by metabolic factors. Maturation-inducing signals play a phenotype-dependent role in the action potential morphology and calcium handling of cells. A microfluidic model was used to investigate the combined effects of mechanical stimulation and metabolic signals on the maturation of hiPSC-CMs^144^. Under stimulation, regardless of the cell source, tissue maturation reduced the variability in the spontaneous beating rate and action potential duration (APD), leading to a more homogeneous cell phenotype.

A model of the human myocardium–microvascular interaction was also developed. King et al. cocultured human hiPSC-CMs with human cardiac microvascular endothelial cells (hCMVECs) and human left ventricular fibroblasts (hLVFBs), as shown in Fig. 5d. Significant electrophysiological modulation, including changes in heart rate, action potentials, calcium handling, and substrates for proarrhythmic alterations, was observed^145^. The coculture was subsequently placed into a microfluidic perfusion system, and vascular self-assembly was subsequently achieved through the infusion of vascular growth factors. A reduction in Ca^2+^ transient peaks and a shortened contraction–relaxation time were observed in the perfused vascularized coculture state, suggesting the modulation of cardiac cell electrophysiology. A microfluidic platform was introduced as a multifunctional and scalable tool for cardiac tissue engineering, as illustrated in Fig. 5e^146^. The platform enabled (i) the 3D self-assembly and growth of cardiac tissue in customizable geometries and orientations, (ii) real-time and parallel detection of contractile stresses exerted by multiple microtissues, and (iii) precise and dynamic control of external mechanical cues.

In addition, the integration of microfluidics with other techniques allows for continuous innovation to meet research demands. FET biosensors integrated into microfluidic chips enhance the immobilization of probes and handle the liquid in the channel^147^. Inspiration from botany facilitated the construction of a plant-driven microfluidic valve system^148^. An automated and independent organ-on-a-chip flow control system achieved constant and pulsatile flow recirculation^149^.

## Summary and outlook

Microfluidic platforms enable high-throughput, multiparameter, and multi-technology integration for the physiological detection of cardiomyocytes. The magnitude and frequency of the contractile force serve as critical mechanical metrics to assess the strength of the beating behavior, reflecting cardiomyocytes’ responses to external stimuli (such as drug stimuli). Electrical metrics include the field potential and action potential. A common parameter for electrical metrics, the field potential, reflects changes in membrane potential intensity during the depolarization and repolarization of cardiomyocytes. In this review, we provide a comprehensive summary of various microfluidic methods for different metrics of cardiomyocytes, categorizing them based on technical approaches. In terms of contractility measurement, this paper first summarizes several common nonmicrofluidic methods for detecting the contraction force, highlighting recent trends and representative achievements during development. For contractility measurements on microfluidic platforms, this paper introduces mainly optical methods. They are capable of combining video and image analysis to monitor the contractility of cardiomyocyte populations with different dimensions and morphologies. Three main methods are used: fluorescence measurement of the intracellular calcium level in single cardiomyocytes to confirm contraction^52^; shifting of the reflection peak of structural color hydrogel films seeded with 2D cardiac tissue for monitoring beating^61^; and image analysis of 3D cardiac tissue loaded on silicon pillars to quantify contractile forces^56^. For the detection of electrophysiology, a common approach is the integration of microfluidics with a microelectrode array (MEA), such as designing groove-patterned layers in a custom microfluidic chip to integrate a commercial MEA system^97^. Recording of field potentials can be achieved for both individual cardiomyocytes and high-throughput measurements in a multiwell plate^92,100^. Electrical stimulation has been widely integrated into electrophysiological devices and extensively applied in microfluidics. The integration of electrical stimulation is beneficial for promoting cell maturation and serves as an effective method to examine the electrical activities of cardiomyocytes.

The significant application of microfluidic platforms in the detection of physiological signals in cardiomyocytes lies in drug screening and disease modeling. Given the high mortality rate of cardiovascular diseases worldwide, drug screening for treatment and modeling of cardiovascular diseases and their related diseases undoubtedly represent research directions that contribute to promoting human health and longevity. Microfluidic platforms are capable of screening drugs and have the ability to replicate the in vivo microenvironment and handle small volumes of liquid. Combined with electromechanical analytical techniques, they can detect signals generated by cardiomyocytes in response to different drug stimuli. This method allows the feasibility of the platform to be evaluated in the application of drug screening, which is intended for future expansion to other cardiac drugs. An important application scenario in this regard is the simultaneous measurement of multiple drugs at various concentrations. The challenge lies in achieving the accuracy and stability of multiple drug tests with multiple concentrations to meet the demands of high throughput. In terms of disease modeling, a microfluidic chip has successfully facilitated the modeling of various types of cardiovascular diseases. Genetic cardiovascular diseases are typically established using iPSC-engineered tissues and patient-derived cells. For nongenetic cardiovascular diseases, microfluidic platforms can simulate the microvascular structure and function to model specific diseases. For example, researchers have investigated thrombus formation^118^, monitored the barrier function of endothelial cells in an atherosclerosis model^98^, and assessed changes in action potential amplitude following myocardial infarction^129^. This information highlights the robust potential of microfluidic platforms in biomimicking the in vivo microenvironment, providing a promising avenue for further exploration in understanding pathogenesis and therapeutic approaches in the biomedical field.

The design strategies of microfluidics have enhanced the physiological study of cardiomyocytes. The purity of cells directly influences subsequent measurements of contractility or electrophysiological signals, whether they are derived from iPSCs or directly obtained from organisms. Microfluidic platforms can enrich cardiomyocytes based on their physical structure or electrophysiological sorting to purify these cells from cell mixtures^137,138^. Multiple strategies have been proposed to facilitate maturation and solve the issue of immature hiPSC-CMs. The application of dynamic loads leads to better cardiac tissue differentiation, as well as electrical and mechanical coupling^143^. The combination of mechanical stimulation and metabolic signals promotes increased maturity and functionality of hiPSC-CMs, enhancing the clinical relevance of drug screening results^144^.

In the future, microfluidics will further advance the field of tissue engineering by replicating and controlling conditions within the cellular microenvironment, influencing the emerging organ-on-a-chip technology^150^. Multiple organs-on-a-chip can interconnect with each other through microfluidics, simulating how they communicate and interact in the body and providing the ability to analyze interactions among organs. Microfluidic platforms that integrate various sensors and interactions between multiple organs can enhance the performance of drug screening, paving the way for the future development of organ-on-a-chip platforms^151^. The coculture of human neurons and cardiomyocytes mimics axon-mediated interactions between them in vivo^152^. Notably, microfluidic platforms can be used for the culture and detection of physiological signals of various cell types. Examples include tumor models of blood and lymphatic vessels for anticancer drug screening^153^, recording of neuronal electrical signals and drug delivery to neurons^154^, and the detection of liver cell secretion^155^.

Digitization and intelligence are remarkable development trends for microfluidic platforms. Digital microfluidics (DMF) is an emerging liquid handling technology based on MEAs for the precise manipulation of discrete droplets. DMF offers the advantages of automation, addressability, integration, and a dynamic configuration and provides a closed reaction space from picoliters to microliters. These properties make it suitable for a laboratory bioanalysis of chips and applications requiring high integration and complex processes^156^. With advancements in mobile computing power, smartphone-based mobile health platforms have drawn significant attention. For example, real-time remote monitoring of cardiac organoids can be achieved through Bluetooth modules to obtain various physiological parameters^157^. Point-of-care testing devices that integrate microfluidic optical detection with artificial intelligence analysis have been developed. The data detected using smartphones can be processed by remote servers for human papillomavirus (HPV) testing^158^. The utilization of deep learning algorithms enables rapid on-site disease diagnosis. In rural areas of South Africa, deep learning algorithms have been applied for rapid classification of images obtained from human immunodeficiency virus (HIV) testing. This approach displayed high sensitivity and specificity while reducing false-positives and false-negatives^159^. Another potential development is the integration of microfluidics with biohybrid robots. Advances in microfluidics have already revolutionized disease modeling and drug development and are positioned to impact regenerative medicine, but have yet to be applied to biohybrids. Fusing microfluidics with living materials will improve tissue perfusion and maturation and enable the precise patterning of sensing, processing, and control elements^160^.
